# Comparative Effectiveness and Safety of Intravenous Versus Oral Iron Therapy With Iron-Deficient Heart Failure: A Network Meta-Analysis of Randomized Controlled Trials

**DOI:** 10.7759/cureus.92087

**Published:** 2025-09-11

**Authors:** Parag N Patel, Devang Mangal, Didar Singh

**Affiliations:** 1 Internal Medicine, GMERS Medical College and Hospital, Dharpur-Patan, Patan, IND; 2 Internal Medicine, Springfield Memorial Hospital, Southern Illinois University, Springfield, USA

**Keywords:** heart failure, iron deficiency anemia (ida), iron infusion, network meta-analysis, oral iron therapy alone

## Abstract

Iron deficiency (ID) is a common and clinically significant comorbidity in patients with heart failure (HF), contributing to reduced exercise capacity, poor quality of life, and increased hospitalization risk. Although intravenous (IV) iron therapy has demonstrated efficacy in improving functional outcomes, the comparative effectiveness of IV vs. oral (per os, or PO) iron supplementation remains uncertain. We conducted a systematic review and network meta-analysis (NMA) of 13 randomized controlled trials (RCTs) evaluating IV iron (ferric carboxymaltose, ferric derisomaltose, iron sucrose), PO iron (ferrous sulfate, ferrous fumarate, polysaccharide, sucrosomial, ferric polymaltose), and placebo in HF patients with ID, analyzed as route-specific class effects. Outcomes analyzed included six-minute walk distance (6MWD), ferritin, transferrin saturation (TSAT), HF hospitalization, all-cause mortality, and cardiovascular (CV) mortality.

We used a Hartung-Knapp random-effects framework with Sidik-Jonkman variance, assessed heterogeneity and inconsistency using I^2^, τ^2^, design-by-treatment interaction, and node-splitting. Risk of bias was assessed independently by two reviewers using Risk of Bias 2 (RoB 2), and certainty of evidence for all outcomes was graded using GRADE adapted for NMA. Because most contrasts included fewer than 10 RCTs, formal tests for publication bias were not feasible, and potential small-study effects were considered qualitatively in the GRADE assessments. Trials that reported outcomes only as medians and interquartile ranges (IQRs), or baseline values without follow-up data, were excluded from quantitative pooling and described narratively.

IV iron significantly improved 6MWD compared to placebo (mean difference (MD) +26.0 m; 95% confidence interval (CI): 18.1 to 33.9), increased ferritin (MD +237.2 μg/L), and reduced the risk of HF hospitalization (risk ratio (RR) 0.79; 95% CI: 0.66 to 0.93), with moderate to high certainty. PO iron showed a comparable, but not statistically significant, mean improvement in 6MWD (MD +35.1 m; 95% CI: -5.2 to +75.4), with wider CIs and inconsistent ferritin and TSAT gains. Neither IV nor PO iron was associated with a significant reduction in all-cause or CV mortality, although a trend toward benefit was observed with IV therapy. Numerical SUCRA values favored IV iron for HF hospitalization (77.9 vs. 57.6 for PO, 14.5 for placebo), ferritin (100.0 vs. 50.0 vs. 0.0), and TSAT (74.0 vs. 75.8 vs. 0.2), while PO iron ranked slightly higher for 6MWD (76.3 vs. 73.7 vs. 0.0). Included PO formulations encompassed both traditional preparations (ferrous sulfate/fumarate, polysaccharide) and newer agents such as sucrosomial iron and ferric polymaltose. Adverse events were comparable across groups: IV iron was not associated with excess mortality or serious adverse events, and PO iron was primarily limited by gastrointestinal intolerance. Sensitivity analyses restricting outcomes to trials with 3-12 months of follow-up showed consistent results, while longer studies mainly influenced event counts rather than the direction of effect. Our findings support the use of IV iron as the preferred strategy to improve symptoms and reduce hospitalizations in HF patients with ID, whereas PO iron may be considered when IV therapy is inaccessible. Further large-scale trials are needed to clarify long-term mortality impact and the role of newer PO formulations.

## Introduction and background

Iron deficiency (ID) is present in up to 50% of heart failure (HF) patients and is associated with worse exercise capacity, poorer quality of life (QOL), and higher hospitalization risk [1]. In HF, ID contributes to skeletal muscle dysfunction and impaired myocardial energetics even before anemia develops [2]. Accordingly, contemporary HF guidelines recommend screening for ID and treating it, especially via intravenous (IV) iron, to improve functional status and HF outcomes [3]. Two large trials (FAIR-HF 2009 and CONFIRM-HF 2015) first established that IV ferric carboxymaltose significantly improves symptoms, six-minute walk distance (6MWD), New York Heart Association (NYHA) class, and QOL in stable HF with reduced ejection fraction (HFrEF) [2,4]. Subsequent trials (including AFFIRM-AHF 2020 and IRONMAN 2022) indicated IV iron may also reduce HF hospitalizations, particularly following acute decompensation [5,6].

In contrast, the role of oral (per os, or PO) iron supplementation in HF remains uncertain. PO iron is inexpensive and widely available, but prior studies (e.g., IRONOUT-HF 2017) did not show improvement in exercise capacity with PO iron polysaccharide, likely due to poor absorption and tolerance in HF patients [7]. Across included randomized controlled trials (RCTs), ID was defined consistently as ferritin <100 µg/L, or 100-300 µg/L with transferrin saturation (TSAT) <20%. Most trials enrolled patients with HFrEF, with limited HFpEF representation. Given the inconvenience and cost of IV therapy, it is important to determine whether newer PO iron formulations or longer therapy might confer benefits, and how IV and PO strategies compare across key outcomes. We focused on 6MWD as a validated measure of functional capacity that correlates with prognosis, and HF hospitalization as a patient-important outcome prioritized in guideline-recommended HF trial endpoints. Safety was analyzed primarily through all-cause and cardiovascular (CV) mortality, with non-mortality adverse events narratively summarized due to sparse and inconsistent reporting.

Recent pairwise and network meta-analyses (NMAs) have reinforced the efficacy of IV iron in reducing HF hospitalizations and possibly CV mortality, though effects on all-cause mortality remain inconclusive [8,9]. A dedicated NMA comparing IV and PO iron in HF reported superior benefit of IV iron for 6MWD and Kansas City Cardiomyopathy Questionnaire (KCCQ) scores, with PO iron showing modest reductions in hospitalization and mortality but inconsistent functional effects [8,9]. However, prior NMAs often underrepresented newer PO formulations, variably reported ranking metrics, and relied on less conservative random-effects estimators. Our study advances the literature by modeling route-specific class effects (IV: ferric carboxymaltose, ferric derisomaltose, iron sucrose; PO: ferrous sulfate/fumarate, polysaccharide, sucrosomial, ferric polymaltose), applying the Hartung-Knapp-Sidik-Jonkman random-effects model with formal inconsistency checks, and grading certainty for all outcomes using the Grading of Recommendations Assessment, Development and Evaluation (GRADE). We also report numerical surface under the cumulative ranking curve (SUCRA) values to complement traditional effect estimates, as SUCRA summarizes the probability that each strategy ranks among the most effective in a multi-treatment comparison.

We included newer PO agents such as sucrosomial iron and ferric polymaltose, which differ pharmacologically from traditional ferrous salts by enhancing gastrointestinal tolerability and absorption. Eligible RCTs spanned 2009-2023, ensuring recency and relevance. Anticipated limitations included heterogeneity in EF phenotypes, dosing and formulation, follow-up duration, and background guideline-directed therapy; these were addressed through random-effects modeling and sensitivity analyses (e.g., restricting to 3-12 months of follow-up).

To address current knowledge gaps, we conducted an updated NMA encompassing 13 RCTs and 6,832 patients with iron-deficient HF. We compared the effects of PO iron, IV iron, and placebo across critical outcomes such as 6MWD, HF hospitalization, all-cause and CV mortality, as well as ferritin and TSAT. Treatments were ranked using SUCRA probabilities, and certainty was assessed via GRADE methodology. Our aim was to clarify the comparative effectiveness and safety of iron supplementation strategies, offering evidence-based guidance for clinical decision-making in iron-deficient HF.

## Review

Methods

Data Sources and Search Strategy

We conducted a comprehensive literature search in PubMed, Cochrane Central Register of Controlled Trials (CENTRAL), and Web of Science for studies published up to 2025. The search combined terms for HF with terms for ID and iron therapy (including “iron deficiency,” “anemia,” “intravenous iron,” “oral iron,” “ferric carboxymaltose,” etc.), without language restrictions. The complete electronic search strategies for each database are provided in Appendix A. In addition, ClinicalTrials.gov and the World Health Organization International Clinical Trials Registry Platform (WHO ICTRP) were checked to confirm eligibility and identify unpublished or ongoing studies. We did not include grey literature, preprints, or conference abstracts, focusing instead on peer-reviewed RCTs. No language restrictions were applied; non-English articles were translated using automated tools and, when necessary, checked by native speakers. We also hand-searched references of relevant reviews and trial registries for additional studies.

**Figure 1:**
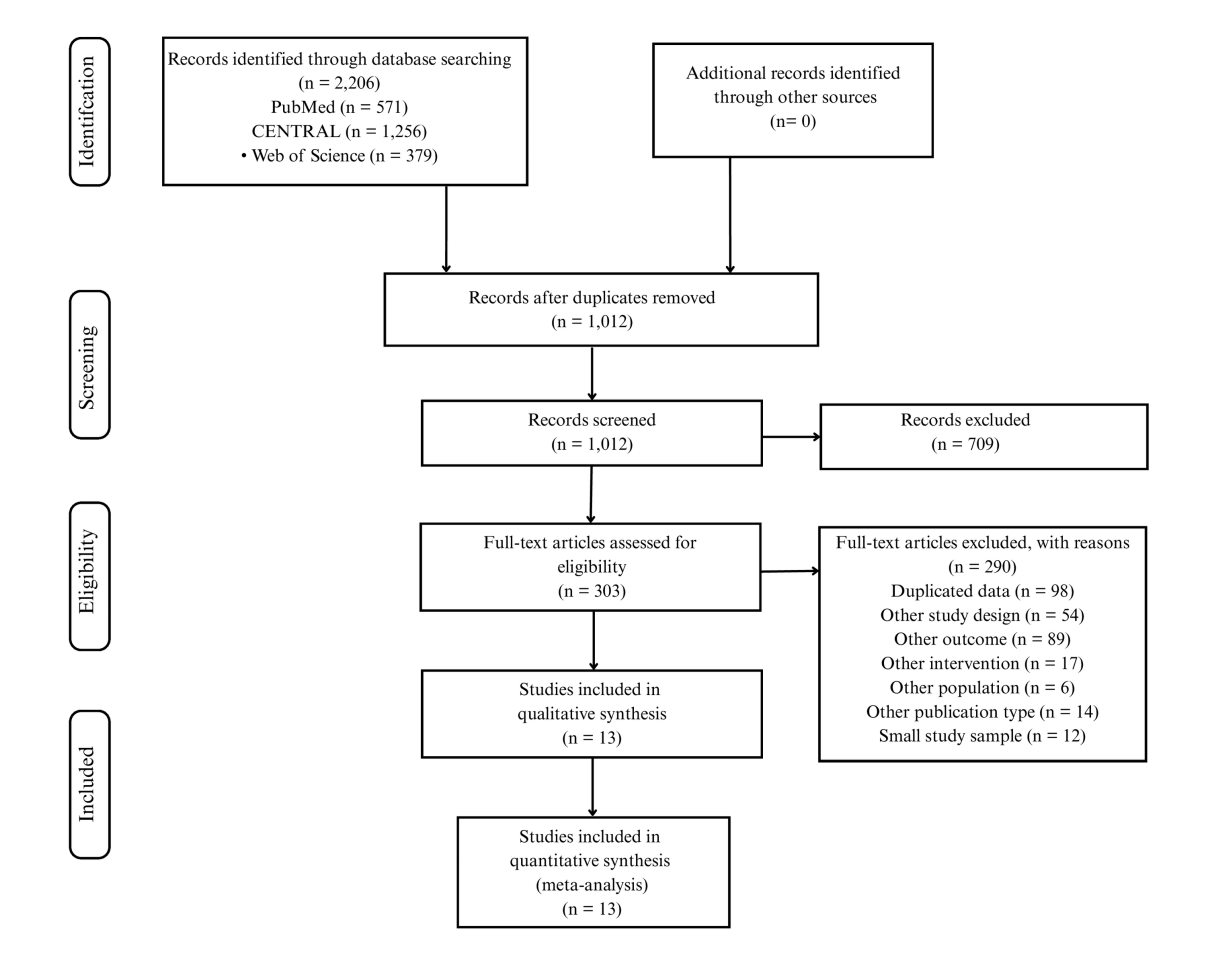
PRISMA flow diagram of study selection. PRISMA: Preferred Reporting Items for Systematic reviews and Meta-Analyses

The initial database search identified 2,206 records (PubMed n = 571; CENTRAL n = 1,256; Web of Science n = 379). After removing duplicates, 1,012 unique records remained. Two reviewers independently screened titles/abstracts, excluding clearly irrelevant papers. Disagreements were resolved by consensus or by a third senior reviewer. A total of 1,012 records were screened, of which 709 were excluded for not meeting the inclusion criteria (e.g., non-HF population or not an RCT). We obtained 303 articles in full text for detailed review. Figure 1 shows the Preferred Reporting Items for Systematic Reviews and Meta-Analyses (PRISMA) flow diagram of study selection. Ultimately, 13 RCTs met the inclusion criteria for qualitative and quantitative synthesis (NMA). The PRISMA 2020 checklist is provided in Appendix B.

Study Selection

We included studies that met the following criteria: (1) RCT (cluster or quasi-randomized designs were not eligible; only true RCTs were included), in adults with HF (any EF category, though all included trials were in HFrEF) and ID; (2) intervention was IV iron therapy or PO iron supplementation, compared to either placebo, standard care, or another form of iron delivery (IV vs. PO); (3) reported at least one relevant outcome (exercise capacity, HF hospitalizations, or mortality, or iron indices); and (4) minimum study duration of 12 weeks. Sensitivity analyses excluding shorter studies confirmed the robustness of results. We required that trials had systematically enrolled HF patients with ID defined by biomarkers (generally ferritin <100 μg/L or 100-300 μg/L with TSAT <20%, as per consensus definition). Trials focusing on acute coronary syndromes or other comorbid populations without separate HF subgroup data were excluded.

Thirteen RCTs satisfied these criteria. These included the seven landmark IV iron trials (FERRIC-HF 2008 [10], FAIR-HF 2009 [2], CONFIRM-HF 2015 [4], EFFECT-HF 2017 [11], AFFIRM-AHF 2020 [5], IRONMAN 2022 [6], and HEART-FID 2023 [12]), one smaller trial of IV vs. PO iron (IRON-HF 2013 [13]), five trials of PO iron vs. placebo (including Sagita at al. 2017 [14], Zdravkovic et al. 2019 [15], IRONOUT-HF 2017 [7], Suryani et al 2016 [16], SI-HF 2021 [17]). Newer PO formulations such as ferric polymaltose (Zdravkovic et al. 2019) and sucrosomial iron (SI-HF 2021) were included and differ pharmacologically from older ferrous salts by improving gastrointestinal tolerability and absorption [15,17]. Where available, adherence and tolerability data (e.g., discontinuation rates, GI adverse events) were collected for descriptive comparison between IV and PO formulations. Table 1 summarizes the key features of these 13 trials. In total, the analysis included data on 6,500+ HF patients (most with HFrEF and NYHA class II-III symptoms, mean left ventricular ejection fraction (LVEF) ~30-40%, moderate ID, and mild anemia) with follow-up ranging from 3 months to 2.7 years. The majority of trials (n = 5) tested IV ferric carboxymaltose (doses guided by body weight and hemoglobin, e.g., 500-1000 mg infusions) vs. placebo; one large trial (IRONMAN) used IV ferric derisomaltose [6]. Five trials evaluated PO iron: one used high-dose PO iron polysaccharide (IRONOUT-HF [7]) and three used other PO formulations (ferrous sulfate in IRON-HF [13], Sagita et al. 2017 [14], Suryani et al. 2016 [16]; PO ferric polymaltose vs. ferrous fumarate in Zdravkovic et al. 2019 [15]; and sucrosomial iron in the SI-HF 2021 [17]). Only one trial (IRON-HF 2013 [13]) directly compared IV vs. PO iron vs. placebo in the same study, underscoring the need for indirect comparisons via NMA (Table 2).

**Table 1:** Inclusion criteria and endpoints of included randomized controlled trials. This table summarizes key demographic and trial-specific inclusion criteria from randomized controlled trials evaluating intravenous or oral iron therapy in patients with heart failure. It includes total sample size, iron deficiency definitions, NYHA functional class, and LVEF inclusion thresholds, follow-up duration, and primary and secondary endpoints. NYHA: New York Heart Association; LVEF: left ventricular ejection fraction; HF: heart failure; TSAT: transferrin saturation; Hb: hemoglobin; 6MWT: 6-minute walk test; VO_2_: oxygen consumption; MLHFQ: Minnesota Living With Heart Failure Questionnaire; KCCQ: Kansas City Cardiomyopathy Questionnaire; PGA: patient global assessment; CV: cardiovascular

Study	Country	Sample Size (Total)	Definition of Iron Deficiency	Inclusion Criteria	Follow-Up Duration	Primary Endpoint	Secondary Endpoint
FERRIC-HF (2008) [10]	UK, Poland, Germany	35	Ferritin <100 μg/L or 100–300 μg/L; TSAT <20%	NYHA II or III (LVEF ≤45%); Peak VO_2_ ≤18 mL/kg/min; Hb ≤12.5 g/dL (anemic) or 12.5-14.5 g/dL (non-anemic)	18 weeks	Change in absolute peak VO_2_ (mL/min)	NYHA class, exercise duration, MLHFQ, fatigue score, TSAT, ferritin, Hb, LVEF, safety
FAIR-HF (2009) [2]	11 countries (Russia, Ukraine, Poland)	459	Ferritin <100 μg/L, or 100–299 μg/L; TSAT <20%	LVEF ≤45%; NYHA II or NYHA III; Hb 95-135 g/L	24 weeks	Patient Global Assessment (PGA); NYHA class at Week 24	6MWT distance, KCCQ score, EQ-5D VAS
CONFIRM-HF (2015) [4]	9 countries (Russia, Ukraine, Poland)	301	Ferritin <100 ng/mL, or 100-300 ng/mL; TSAT <20%	NYHA II or III (LVEF ≤45%); BNP >100 pg/mL or NT-proBNP >400 pg/mL; Hb <15 g/dL	52 weeks	Change in 6-minute walk test (6MWT) at 24 weeks	NYHA class, PGA, fatigue score, QoL scores (KCCQ, EQ-5D), HF hospitalizations, death
EFFECT-HF (2017) [11]	9 countries (NR)	174	Ferritin <100 ng/mL, or 100-300 ng/mL; TSAT <20%	NYHA II–III (LVEF ≤45%); Peak VO_2_ 10-20 mL/kg/min; BNP >100 pg/mL or NT-proBNP >400 pg/mL	24 weeks	Change in peak VO_2_ (mL/kg/min)	TSAT, ferritin, Hb, BNP/NT-proBNP, NYHA class, PGA, VE/VCO_2_ slope, hospitalizations, safety
AFFIRM-AHF (2020) [5]	15 countries (Georgia, Ukraine, Romania)	1108	Ferritin <100 ng/mL, or 100-299 ng/mL; TSAT <20%	Hospitalized for acute HF; LVEF <50%; Received ≥40 mg IV furosemide; NT-proBNP ≥1600 pg/mL (SR) or ≥2400 pg/mL (AF); Hb 80-150 g/L	52 weeks	Composite of total HF hospitalizations and cardiovascular death	Total CV hospitalizations + CV death, CV death alone, time to first HF hospitalization or CV death, days lost due to HF hospitalizations or CV death
IRONMAN (2022) [6]	UK	1137	Ferritin <100 µg/L or TSAT <20%	LVEF ≤45% within prior 24 months; Current or recent (<6 m) HF hospitalization; NT-proBNP>250 pg/mL (SR) or >1000 pg/mL (AF); Hb: Women 90-130 g/L and men 90-140 g/L	Median 2.7 years (IQR 1.8-3.6)	Composite of recurrent HF hospitalizations and cardiovascular death	CV hospitalization (first event), CV death, MLHFQ and EQ-5D scores, 6MWT (4 and 20 mo), all-cause mortality, HF rehospitalization, safety endpoints
HEART-FID (2023) [12]	14 countries (North America, EU, Russia/Ukraine)	3065	Ferritin <100 ng/mL, or 100-300 ng/mL; TSAT <20%	HF with LVEF ≤40%; Hb >9 g/dL and <13.5 (F) or <15 (M)	Median 1.9 (IQR 1.3-3.0)	Hierarchical composite of 1) Death at 12 months, 2) HF hospitalization at 12 months, 3) Change in 6MWD at 6 months	CV death or HF hospitalization, MLHFQ, EQ-5D
IRON-HF (2013) [13]	Brazil	23	TSAT <20% and ferritin <500 μg/L	Stable outpatients with HF for ≥3 months; NYHA II-IV; LVEF <40%; Hb 9-12 g/dL	12 weeks	Change in peak VO_2_ (mL/kg/min)	Hb increase, TSAT >20%, anemia correction, ferritin change
Sagita et al. (2017) [14]	Jakarta, Indonesi		Ferritin 100 μg/L OR 100-300 μg/L; TSAT <20%	LVEF <50%	12 weeks	Global longitudinal train	-
Zdravkovic et al. (2019) [15]	Nis, Serbia	201	Serum ferritin < 100 µg/L and TSAT < 20%	CHF patients with anemia (Hb <13 g/dL in men, <12 g/dL in women per WHO)	24 weeks	Death or rehospitalization due to cardiac causes	Change in 6-minute walk test (6MWT), LVEF, hemoglobin, and tolerability of iron therapy
IRONOUT-HF (2017) [7]	US	225	Ferritin 15-100 ng/mL or 100-299 ng/mL; TSAT <20%	HFrEF with LVEF ≤40%; NYHA II–IV symptoms; Hemoglobin: 9-15 g/dL (men), 9-13.5 g/dL (women)	16 weeks	Change in peak VO_2_ (mL/kg/min)	6MWT, NT-proBNP, KCCQ, iron indices, hepcidin, and sTfR changes
Suryani et al. 2016 [16]	Indonesia	54	Ferritin <100 ng/mL or 100-300 ng/mL; TSAT <20%	HFrEF (EF <45%) with NYHA II-III; Hb <13 g/dL (men), <12 g/dL (women)	12 weeks	Change in 6MWT	LVEF, iron profile, clinical outcomes
SI-HF (2021) [17]	Italy	50	Ferritin <100 ng/mL or 100-299 ng/mL; TSAT <20%	HFrEF (LVEF <40%) with symptoms; on stable guideline-directed therapy for ≥4 weeks	24 weeks	Change in 6MWD after 3 months	KCCQ score, NT-proBNP, HF hospitalization, urgent HF visits

**Table 2:** Baseline clinical parameters of trial participants by treatment arm. Parameters include age, NYHA class distribution, hemoglobin levels, ferritin concentration, transferrin saturation (TSAT), and BNP or NT-proBNP values. Values are presented as reported in the original trial publications (mean ± SD or median (IQR)); no conversions were performed. NYHA: New York Heart Association; Hb: hemoglobin; BNP: B-type natriuretic peptide; NT-proBNP: N-terminal pro B-type natriuretic peptide; FCM: ferric carboxymaltose; IV: intravenous

Study	Arm	Sample Size (Per Arm)	Iron Form/Class	Age (Mean ± SD)	NYHA Class Distribution (I/II/III/IV)	Hemoglobin (g/dL)	Ferritin (ng/mL)	TSAT (%)	BNP or NT-proBNP Criteria
FERRIC-HF (2008) [10]	IV Iron	24	IV Iron Sucrose	64 ± 14 years	0/13/11/0	≤12.5 g/dL	<100 or 100-300	<20%	Not reported
	Placebo	11		62 ± 11 years	0/6/5/0	12.5-14.5 g/dL	<100 or 100-300 <20%	<20%	Not reported
FAIR-HF (2009) [2]	IV Iron	304	IV FCM	67.8 ± 10.3 years	0/53/251/0	11.9 ± 1.3	52.5 ± 54.5	17.7 ± 12.6	Not reported
	Placebo	155		67.4 ± 11.1 years	0/29/126/0	11.9 ± 1.4	60.1 ± 66.5	16.7 ± 8.4	Not reported
CONFIRM-HF (2015) [4]	IV Iron	150	IV FCM	68.8 ± 9.5 years	0/80/70/0	12.37 ± 1.41	57.0 ± 48.4	20.2 ± 17.6	BNP >100 pg/mL or NT-proBNP >400 pg/mL (Inclusion criteria)
	Placebo	151		69.5 ± 9.3 years	0/91/60/0	12.42 ± 1.30	57.1 ± 41.6	18.2 ± 16.1	BNP >100 pg/mL or NT-proBNP >400 pg/mL (Inclusion criteria)
EFFECT-HF (2017) [11]	IV Iron	88	IV FCM	63 ± 12 years	0/61/25/0	12.9 ± 1.3	48 (median)	17.3 (median)	BNP >100 pg/mL or NT-proBNP >400 pg/mL (Inclusion criteria)
	Placebo	86		64 ± 11 years	0/54/32/0	13.0 ± 1.5	53 (median)	18.1 (median)	BNP >100 pg/mL or NT-proBNP >400 pg/mL (Inclusion criteria)
AFFIRM-AHF (2020) [5]	IV Iron	567	IV FCM	71.2 ± 10.8 years	14/255/277/6	12.3 ± 1.6	83.9 ± 62.2	15.2 ± 8.3	BNP median: 1068 pg/mL NT-proBNP median: 4743 pg/mL (IQR 2781-8128)
	Placebo	565		70.9 ± 11.1 years	8/240/276/22	12.1 ± 1.6	88.5 ± 68.8	14.2 ± 7.5	BNP median: 1204 pg/mL NT-proBNP median: 4684 pg/mL (IQR 2785-8695)
IRONMAN (2022) [6]	IV Iron	569	IV Ferric Derisomaltose	73.2 (66.7-80.1) (Median (IQR))	0/328/230/11	12.1 (IQR 11.2-12.8) (Median)	49.0 (30.0-86.0) (median)	15% (11-20) (median)	NT-proBNP >250 (SR) or >1000 (AF), BNP >75 (SR) or >300 (AF) (Inclusion criteria)
	Placebo	568		73.5 (67.1-79.1) (Median (IQR))	0/320/238/10	12.1 (IQR 11.2-12.9) (Median)	50.0 (30.0-85.0) (median)	15% (10-19) (median)	NT-proBNP >250 (SR) or >1000 (AF), BNP >75 (SR) or >300 (AF) (Inclusion criteria)
HEART-FID (2023) [12]	IV Iron	1532	IV FCM	68.6 ± 10.9 years	0/797/711/22	12.6 ± 1.4	56.0 ± 47.3	23.9 ± 11.2	NT-proBNP median: 1485.5 (IQR: 727.1-3044.5)
	Placebo	1533		68.6 ± 11.2 years	0/820/692/19	12.5 ± 1.4	12.5 ± 1.4	23.0 ± 10.3	NT-proBNP median: 1423.6 (IQR: 710.0-2883.8)
IRON-HF (2013) [13]	IV Iron	10	IV Iron Sucrose	66.9 ± 8.3	Not reported	11.2 ± 0.6	185 ± 146	18.9 ± 9.7	Not reported
	PO Iron	7	PO Ferrous Sulfate	63.5 ± 16.2	Not reported	11.3 ± 0.5	101 ± 135	18.8 ± 8.6	Not reported
	Placebo	6		68.9 ± 10.1	Not reported	10.9 ± 0.7	95 ± 128	13.5 ± 5.8	Not reported
Sagita et al. (2017) [14]	PO Iron	Total 37	PO Ferrous Sulfate	Not reported	Not reported	≤13 g/dL	<100 or 100-300	<20%	Not reported
	Placebo	Total 37		Not reported	Not reported	≤13 g/dL	<100 or 100-300 <20%	<20%	Not reported
Zdravkovic et al. (2019) [15]	PO Iron	100	PO Ferrous Fumarate	73.31 ± 9.77 years	0/45/49/6	11.5 ± 1.1	8.66 ± 4.15 (Mild ID at entry)	8.7 ± 4.1	BNP (pg/mL) 1163.07 ± 1213.02
	PO iron	101	PO Ferric Hydroxide Polymaltose Complex	70.76 ± 9.81 years	0/37/56/8	11.8 ± 1.2	8.52 ± 3.91 (Mild ID at entry)	8.5 ± 3.9	BNP (pg/mL) 1141.37 ± 1153.66
IRONOUT-HF (2017) [7]	PO IRON	111	PO Iron Polysaccharide	63.0 (54.0-71.0) (Median (IQR))	0/81/30/0	12.6 (11.7-13.3) (Median (IQR))	75 (43-108) (Median (IQR))	19 (16-24)(Median (IQR))	BNP (pg/mL) 1072 (413-2286) (Median (IQR))
	Placebo	114		63.0 (55.0-70.0) (Median (IQR))	0/69/45/0	12.7 (11.8-13.4) (Median (IQR))	70 (42-111) (Median (IQR))	19 (16-24) (Median (IQR))	BNP (pg/mL) 1170 (527-2530) (Median (IQR))
Suryani et al. (2016) [16]	PO Iron	27	PO Ferrous Sulphate	58 ± 9 years	0/11/11/0	11.6 ± 1.8	121 ± 108	15.6 ± 5	NT-proBNP (pg/mL) 2810 ± 3116
	Placebo	27		57 ± 10 years	0/11/8/0	11.3 ± 1.0	110 ± 72	17 ± 7.6	NT-proBNP (pg/mL) 3105 ± 2354
SI-HF (2021) [17]	PO Iron	25	PO Sucrosomial Iron	69 ± 8 years	0/15/10/0	12.5 ± 1.1	38.8 ± 24.3	<20%	BNP (pg/mL) 462 ± 529
	Placebo	25		69 ± 8 years	0/14/11/0	12.9 ± 0.9	44.6 ± 31.7	<20%	BNP (pg/mL) 350 ± 308

Risk of Bias Assessment

**Figure 2:**
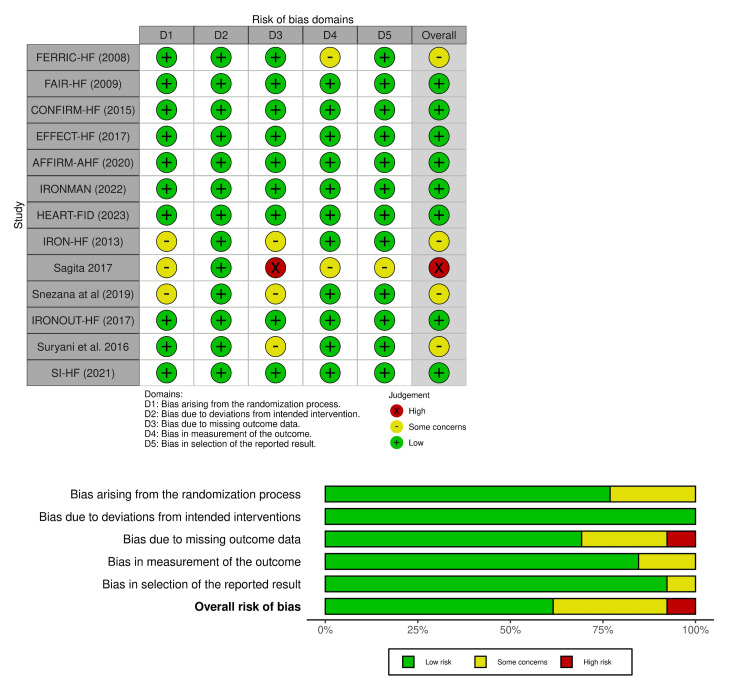
Risk of bias assessment across included studies. Traffic-light plot showing individual study-level risk of bias assessments using the Cochrane Risk of Bias 2.0 (RoB 2.0) tool, along with a domain-level summary of risk of bias judgments across the 13 randomized controlled trials (RCTs). Risk of bias assessment using the revised Cochrane RoB 2.0 tool [18]. References [10,2,4,11,5,6,12-15,7,16,17]

Risk of bias was assessed for each trial using the Cochrane Risk of Bias 2.0 (RoB 2.0) tool [18]. Two reviewers independently judged each domain as “low risk,” “some concerns,” or “high risk,” with disagreements resolved by consensus or, if needed, by a third reviewer. The assessment was performed after data extraction but before evidence synthesis. A summary is shown in Figure 2. Overall, most multicenter RCTs were low risk, and sensitivity analyses excluding high-risk trials did not materially alter the results. Expanded criteria, thresholds, and inter-rater reliability details are provided in Appendix C.

Outcomes and Data Extraction

We extracted data on all relevant outcomes reported in the trials. The prespecified outcomes for our review were exercise capacity measured by 6MWD (in meters); iron indices: change in serum ferritin (μg/L) and TSAT (%) from baseline; clinical endpoints: all-cause mortality, CV mortality, and HF hospitalizations (generally defined as worsening HF requiring inpatient treatment).

Outcomes considered (for descriptive purposes) included NYHA functional class improvement and quality-of-life scores (e.g., KCCQ), though these were not meta-analyzed due to insufficient data.

Two reviewers independently extracted outcome data at the longest follow-up available (6-12 months for most trials, and up to 2.7 years in IRONMAN [6]). For continuous outcomes (6MWD, ferritin, TSAT), we recorded the mean change from baseline in each arm and the corresponding standard deviation (SD). For binary outcomes (all-cause mortality, CV deaths, and HF hospitalization), we extracted the number of patients with events and total N per arm. In trials reporting time-to-event outcomes (e.g., recurrent HF hospitalizations), we used hazard ratios if available; however, for consistency, the main analysis used simple risk ratios (RRs) at trial end (since few trials had time-to-event data). Trials reporting only baseline or median/interquartile range (IQR) values without mean change and SDs were excluded from quantitative pooling and described narratively. When partial data (e.g., SDs) were missing, we attempted imputation from available statistics; if not feasible, the trial was excluded from that outcome.

Data consistency was checked, and any discrepancies or ambiguities were resolved by consulting the original publication or contacting authors if needed. For NMA, we focused on the three interventions of interest: IV iron, PO iron, and placebo/standard care. Where a trial had multiple intervention arms (e.g., IRON-HF had IV, PO, and placebo arms; the Serbian trial had two different PO iron formulations), we combined arms or selected the most relevant comparison to fit a three-node network (for the Serbian study, we combined the two PO iron arms as a single PO iron node, assuming class effect). This approach preserves randomization while allowing a connected network of comparisons.

Data Synthesis and Statistical Analysis

We performed random-effects network and pairwise meta-analyses using the Hartung-Knapp-Sidik-Jonkman (HK-SJ) method, implemented via the meta and netmeta packages in R (R Foundation for Statistical Computing, Vienna, Austria). The network geometry included three nodes: IV iron, PO iron, and placebo. Effect measures were mean differences (MDs) for continuous outcomes and RRs for dichotomous outcomes, each with 95% confidence intervals (CIs). Pairwise comparisons were evaluated first, followed by an NMA synthesizing direct and indirect evidence.

Transitivity was assumed on the basis of comparable trial populations (predominantly HFrEF with ID defined by ferritin/TSAT criteria) and interventions. A common heterogeneity parameter was applied across comparisons. Multi-arm trials were managed by combining relevant arms at the node level to preserve randomization and avoid double-counting.

Heterogeneity was summarized using I^2^ and τ^2^ in pairwise analyses, and global and local inconsistency in the network was assessed using the design-by-treatment interaction test and node-splitting. Because most contrasts involved fewer than 10 trials, formal Egger’s or Peters’ tests were not feasible; small-study effects and potential publication bias were considered qualitatively within the GRADE framework. Sensitivity analyses using fixed-effect vs. random-effects models and excluding high-risk-of-bias trials did not materially alter the findings.

Ranking of treatments: SUCRA values were calculated for all outcomes (6MWD, ferritin, TSAT, HF hospitalization, all-cause mortality, CV mortality). Rankings were interpreted alongside effect estimates and certainty ratings, not as definitive evidence of clinical superiority.

All analyses were conducted in R (netmeta package), following PRISMA-NMA guidelines. The PRISMA flow diagram is shown in Figure 1, and the PRISMA 2020 checklist is provided in Appendix B. The protocol for this systematic review was registered with the International Prospective Register of Systematic Reviews (PROSPERO) (CRD4201110444). Additional methodological details, including normality checks, handling of missing data, harmonization of follow-up, and prediction intervals, are provided in Appendix D.

Certainty of Evidence

We evaluated the certainty of evidence for each key outcome using the GRADE framework, adapted for NMA [19]. This involved assessing risk of bias, inconsistency, indirectness, imprecision, and publication bias. For each comparison of interest (IV vs. placebo, PO vs. placebo, IV vs. PO), we started at high certainty (for RCT evidence) and downgraded as needed. Certainty was assessed for all outcomes, including 6MWD, HF hospitalization, all-cause mortality, CV mortality, ferritin, and TSAT.

Results

Included Studies and Patient Characteristics

Key characteristics of the 13 included trials are summarized in Table 1. The trials were published between 2008 and 2023, reflecting evolving interest in iron therapy in HF. Sample sizes ranged from 23 patients to 3,065. All trials enrolled patients with HFrEF (EF typically <45%) and ID per standard definitions. Mean baseline ferritin levels were often in the 50-100 μg/L range, TSAT around 15%, and hemoglobin in the 11-13 g/dL range (most patients were not severely anemic). Patients were generally NYHA class II-III, on guideline-directed HF therapies (see Table 2 for details). Outcome reporting across included trials is summarized in Table 3. This matrix outlines which outcomes were available in each study, helping to contextualize the contribution of each RCT to the different comparisons made in the NMA.

**Table 3:** Outcome reporting matrix across included randomized controlled trials. 6MWD: six-minute walk distance; NYHA: New York Heart Association; HF: heart failure; CV: cardiovascular; TSAT: transferrin saturation

Study	6MWD	NYHA Class	HF Hospitalization	All-Cause Mortality	CV Deaths	Ferritin	TSAT
FERRIC-HF (2008) [10]	Not reported	Reported	Reported	Reported	Reported	Reported	Reported
FAIR-HF (2009) [2]	Reported	Reported	Reported	Reported	Reported	Reported	Reported
CONFIRM-HF (2015) [4]	Reported	Not reported	Reported	Reported	Reported	Only baseline reported	Only baseline reported
EFFECT-HF (2017) [11]	Not reported	Not reported	Reported	Reported	Reported	Reported	Reported
AFFIRM-AHF (2020) [5]	Not reported	Not reported	Reported	Reported	Reported	Not reported	Not reported
IRONMAN (2022) [6]	Reported	Reported	Reported	Reported	Reported	Reported as median only	Reported as median only
HEART-FID (2023) [12]	Reported	Not reported	Reported	Reported	Reported	Reported	Reported
IRON-HF (2013) [13]	Not reported	Reported	Not reported	Reported	Reported	Reported	Reported
Sagita et al. (2017) [14]	Only baseline reported	Not reported	Reported	Reported	Not reported	Only baseline reported	Only baseline reported
Zdravkovic et al. (2019) [15]	Only baseline reported	Not reported	Reported	Reported	Not reported	Reported	Only baseline reported
IRONOUT-HF (2017) [7]	Reported as median only	Not reported	Not reported	Reported	Reported	Reported as median only	Reported as median only
Suryani et al. (2016) [16]	Reported	Not reported	Reported	Reported	Not reported	Reported	Reported
SI-HF (2021) [17]	Reported	Not reported	Reported	Reported	Reported	Reported	Not reported

Six-Minute Walk Distance (6MWD)

**Figure 3:**
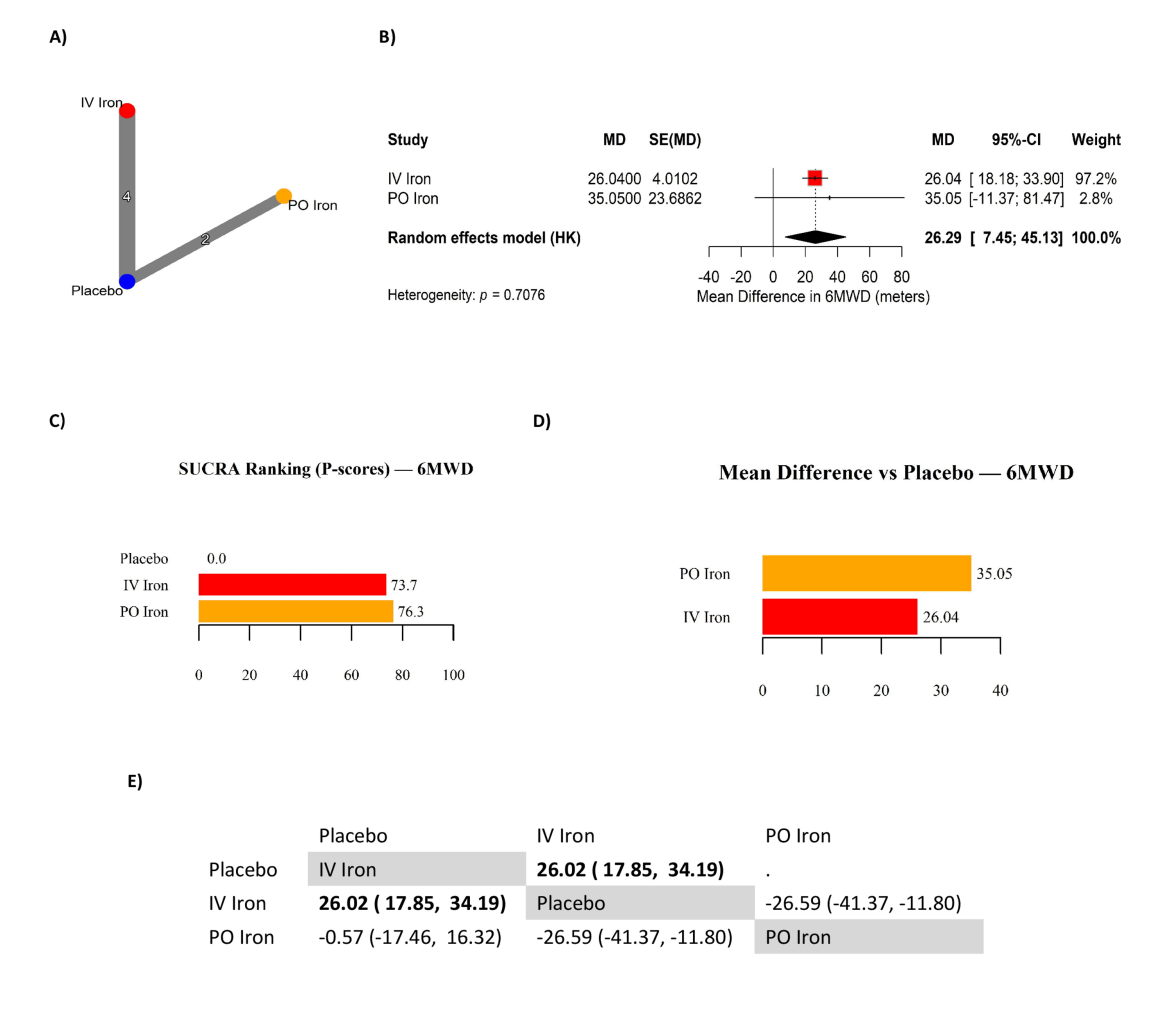
Network meta-analysis of six-minute walk distance (6MWD) in patients with iron-deficient heart failure. (A) Network geometry. (B) Forest plot displaying the pooled mean difference (MD). (C) Surface under the cumulative ranking curve (SUCRA; P-score) rankings. (D) Horizontal bar plot showing direct MDs. (E) League table of pairwise MDs with 95% confidence intervals (CI). Mean difference in 6MWD (meters). References [2,4,6,12,15,7,16,17] IV: intravenous; PO: per os (oral)

A total of six RCTs contributed to the NMA evaluating the effect of PO and IV iron therapy on functional capacity, measured by the 6MWD, encompassing 5,495 patients (Table 4). The network structure demonstrated adequate connectivity via placebo-controlled trials, though no direct head-to-head comparisons were available between IV and PO iron (Figure 3A). Compared to placebo, IV iron significantly improved 6MWD with a pooled MD of 26.04 meters (95% CI: 18.13 to 33.90), representing a consistent benefit across studies (Figure 3B). PO iron showed a numerically larger effect (MD: 35.05 meters; 95% CI: -11.37 to 81.47), but the estimate was imprecise and not statistically significant due to limited sample size and wide variance. The overall random-effects estimate across all interventions confirmed a significant improvement in functional capacity (MD: 26.29 meters; 95% CI: 7.45 to 45.13), with no evidence of significant heterogeneity (Q test p = 0.71) (Figure 3D). Ranking probabilities based on SUCRA values suggested a slight numerical advantage for PO iron (76.3) over IV iron (73.7), with placebo ranking lowest (0.0) (Figure 3C). However, this discrepancy reflects the imprecision of PO iron estimates: the statistically significant indirect network contrast demonstrated an advantage of IV iron over PO iron (MD: 26.59 meters; 95% CI: -41.37 to -11.80) (Figure 3E). Thus, although SUCRA values numerically favored PO iron, IV iron provided more precise and consistent benefit across studies. Follow-up durations ranged from 12 to 52 weeks. Restricting analyses to trials with 3-12 months of follow-up yielded similar results, with longer-duration trials primarily influencing precision rather than direction of effect. Between-arm baseline imbalances in 6MWD were minimal (<10 m across all trials), and the pooled MD values therefore reflect between-arm differences at follow-up rather than within-arm changes. Overall, the evidence supports IV iron as the more reliable intervention for improving 6MWD in iron-deficient HF, with stronger precision and consistency compared to PO iron. The observed ~26 m improvement exceeds the minimal clinically important difference (MCID) of ~20-25 m for HF populations, supporting its clinical as well as statistical significance.

**Table 4:** Six-minute walk distance (6MWD) outcomes across included trials. This table summarizes baseline and follow-up 6MWD values by treatment arm in randomized controlled trials evaluating intravenous and PO iron therapy in patients with heart failure. Data include sample size (N), iron class used, baseline and final 6MWD (in meters), mean change in 6MWD (Δ6MWD) with standard deviation (SD), and follow-up duration. When reported, values are presented as mean ± SD. Studies with both IV and PO iron formulations are included, allowing arm-wise comparisons to placebo or active control. IV: intravenous; PO: per os (oral); FCM: ferric carboxymaltose; NR: not reported

Study	Arm	Iron Class	N	Baseline 6MWD (m)	Final 6MWD (m)	Δ6MWD (Mean ± SD)	Follow-Up
FAIR-HF (2009) [2]	IV Iron	IV FCM	304	274 ± 6	313 ± 7	39 ± 9	24 weeks
	Placebo		155	269 ± 10	277 ± 10	8 ± 10	24 weeks
CONFIRM-HF (2015) [4]	IV Iron	IV FCM	152	288 ± 98	306 ± 98	18 ± 8	52 weeks
	Placebo		152	302 ± 97	286 ± 97	-16 ± 8	52 weeks
IRONMAN (2022) [6]	IV Iron	IV Ferric Derisomaltose	569	274 ± 6	313 ± 7	35 ± 8	24 weeks
	Placebo		568	287.7 ± 9.6	288.8 ± 13.9	1.1 ± 16.88	24 weeks
HEART-FID (2023) [12]	IV Iron	IV FCM	1532	273.9 ± 109.7	NR	8 ± 60	24 weeks
	Placebo		1533	274.7 ± 109.4	NR	4 ± 59	24 weeks
Zdravkovic et al. (2019) [15]	PO Iron	PO Ferrous Fumarate	100	376.1 ± 125.73	438.17 ± 113.09	62.07	24 weeks
	PO Iron	PO Ferric Hydroxide Complex	101	299.75 ± 121.90	335.13 ± 93.15	35.38	24 weeks
IRONOUT-HF (2017) [7]	PO Iron	PO Polysaccharide Iron	111	63.0 (54.0-71.0) (Median (IQR))	366 (315-456) (Median (IQR))	19 (-19 to 51) (Median (IQR))	16 weeks
	Placebo		114	63.0 (55.0-70.0) (Median (IQR))	397 (299-472) (Median (IQR))	32 (-12 to 66) (Median (IQR))	16 weeks
Suryani et al. 2016 [16]	PO Iron	PO Ferrous Sulfate	27	300 ± 85	349 ± 86	46.23 ± 35	12 weeks
	Placebo		27	309 ± 75	305 ± 85	-13.7 ± 46	12 weeks
SI-HF (2021) [17]	PO Iron	PO Sucrosomial Iron	25	332 ± 78	NR	9.50 ± 15.83	24 weeks
	Placebo		25	333 ± 78	NR	3.00 ± 8.81	24 weeks

HF Hospitalizations

**Figure 4:**
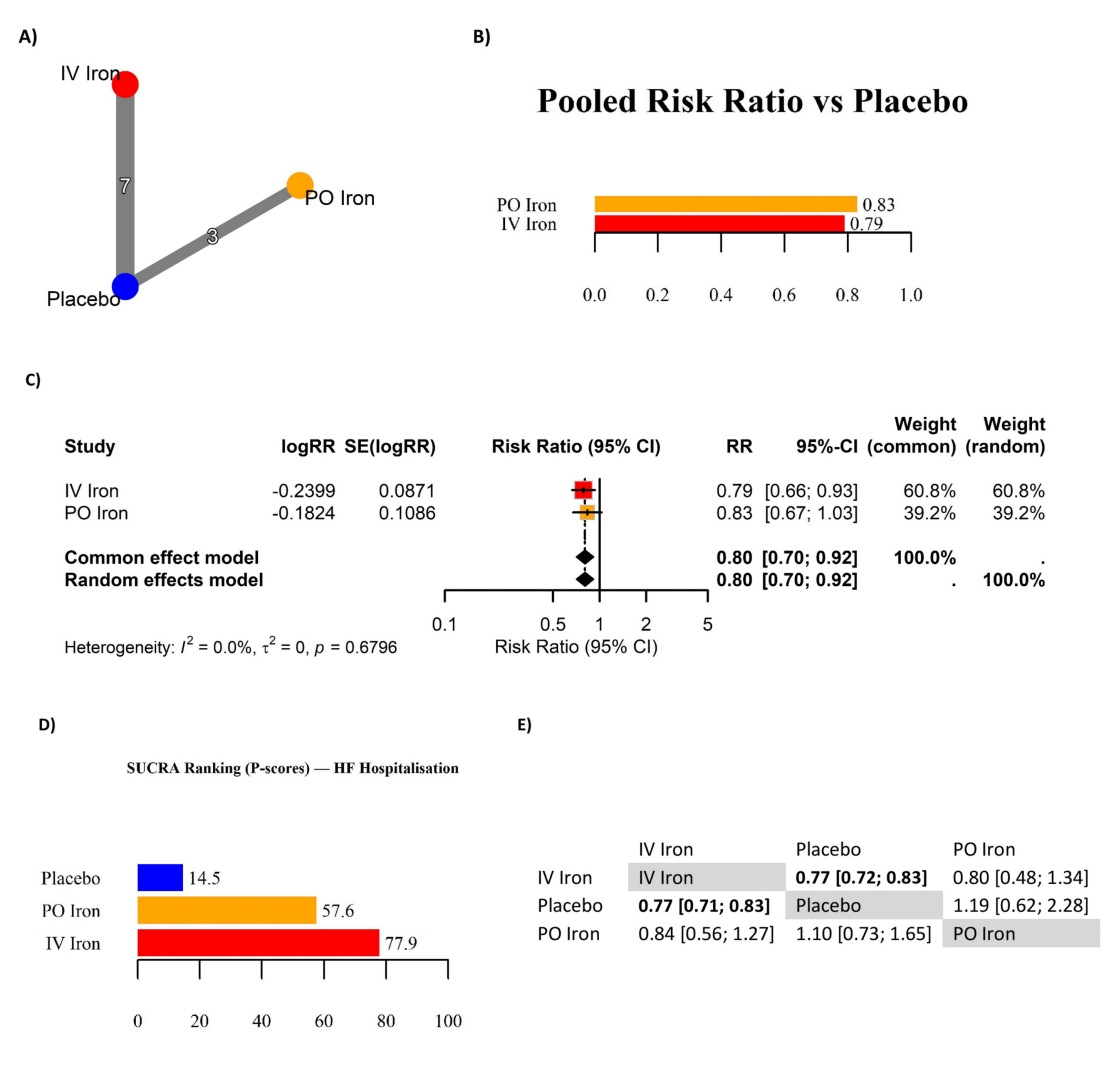
Network meta-analysis of heart failure hospitalizations in patients with iron-deficient heart failure. (A) Network geometry. (B) Pooled risk ratio (RR) versus placebo. (C) Forest plot of RRs. (D) Surface under the cumulative ranking curve (SUCRA; P-scores) rankings for heart failure hospitalization. (E) League table of pairwise RRs. References [10,2,4,11,5,6,12,15-17] HF: heart failure; IV: intravenous; PO: per os (oral); CI: confidence interval

Ten RCTs were included in the NMA evaluating the effect of iron therapy on the risk of HF hospitalization (Table 5). The network geometry (Figure 4A) was well-connected through seven IV iron vs. placebo and three PO iron vs. placebo comparisons, though no trials directly compared IV and PO iron. Both IV and PO iron therapies demonstrated a favorable reduction in HF hospitalization risk compared to placebo. Pooled estimates showed a statistically significant reduction with IV iron (RR: 0.79, 95% CI: 0.66 to 0.93) and a non-significant effect with PO iron (RR: 0.83, 95% CI: 0.67 to 1.03) (Figure 4B). The overall random-effects pooled estimate across both interventions was significant (RR: 0.80, 95% CI: 0.70 to 0.92), with no evidence of heterogeneity (I^2^ = 0%, p = 0.68), supporting consistency across trials (Figure 4C). SUCRA rankings (Figure 4D) placed IV iron highest in probability of being the most effective treatment (77.9), followed by PO iron (57.6) and placebo (14.5). This ranking difference should be interpreted cautiously, as PO iron estimates were imprecise with wide CIs. The statistically significant advantage of IV iron over placebo was confirmed in the indirect network contrast, whereas PO iron did not achieve significance against either comparator (Figure 4E). Analyses were based on first hospitalization events as reported by the trials; recurrent event models (e.g., Andersen-Gill, negative binomial) were not available across all studies and therefore not pooled. Follow-up duration varied from 12 weeks to 2.7 years. Restricting analyses to studies with 3-12 months of follow-up confirmed a consistent direction of effect, with longer trials mainly influencing event counts rather than effect size. No trial arm reported zero HF hospitalization events, so continuity corrections were not required. Sensitivity analyses excluding the smaller PO iron studies did not materially change the pooled IV iron effect, and IV iron consistently ranked highest in probability of benefit. Taken together, these results support IV iron as the most reliable intervention for reducing HF hospitalizations, with consistent efficacy across trials and higher precision than PO iron.

**Table 5:** Heart failure hospitalization events by treatment arm in included trials. HF: heart failure; IV: intravenous; PO: per os (oral)

Study	Arm	N	HF Hospitalizations (n)	Follow-Up
FERRIC-HF (2008) [10]	IV Iron	24	1	18 weeks
	Placebo	11	1	18 weeks
FAIR-HF (2009) [2]	IV Iron	304	7	24 weeks
	Placebo	155	9	24 weeks
CONFIRM-HF (2015) [4]	IV Iron	152	10	52 weeks
	Placebo	152	32	52 weeks
EFFECT-HF (2017) [11]	IV Iron	88	13	24 weeks
	Placebo	86	13	24 weeks
AFFIRM-AHF (2020) [5]	IV Iron	567	293	52 weeks
	Placebo	565	372	52 weeks
IRONMAN (2022) [6]	IV Iron	569	250	Median ~2.7 years (IQR 1.8-3.6)
	Placebo	568	313	Median ~2.7 years (IQR 1.8-3.6)
HEART-FID (2023) [12]	IV Iron	1532	297	52 weeks
	Placebo	1533	332	52 weeks
Zdravkovic et al. (2019) [15]	PO Iron	100	10	24 weeks
	Placebo	101	11	24 weeks
Suryani et al. 2016 [16]	PO Iron	27	1	12 weeks
	Placebo	27	1	12 weeks
SI-HF (2021) [17]	PO Iron	25	4	24 weeks
	Placebo	25	6	24 weeks

All-Cause Mortality

**Figure 5:**
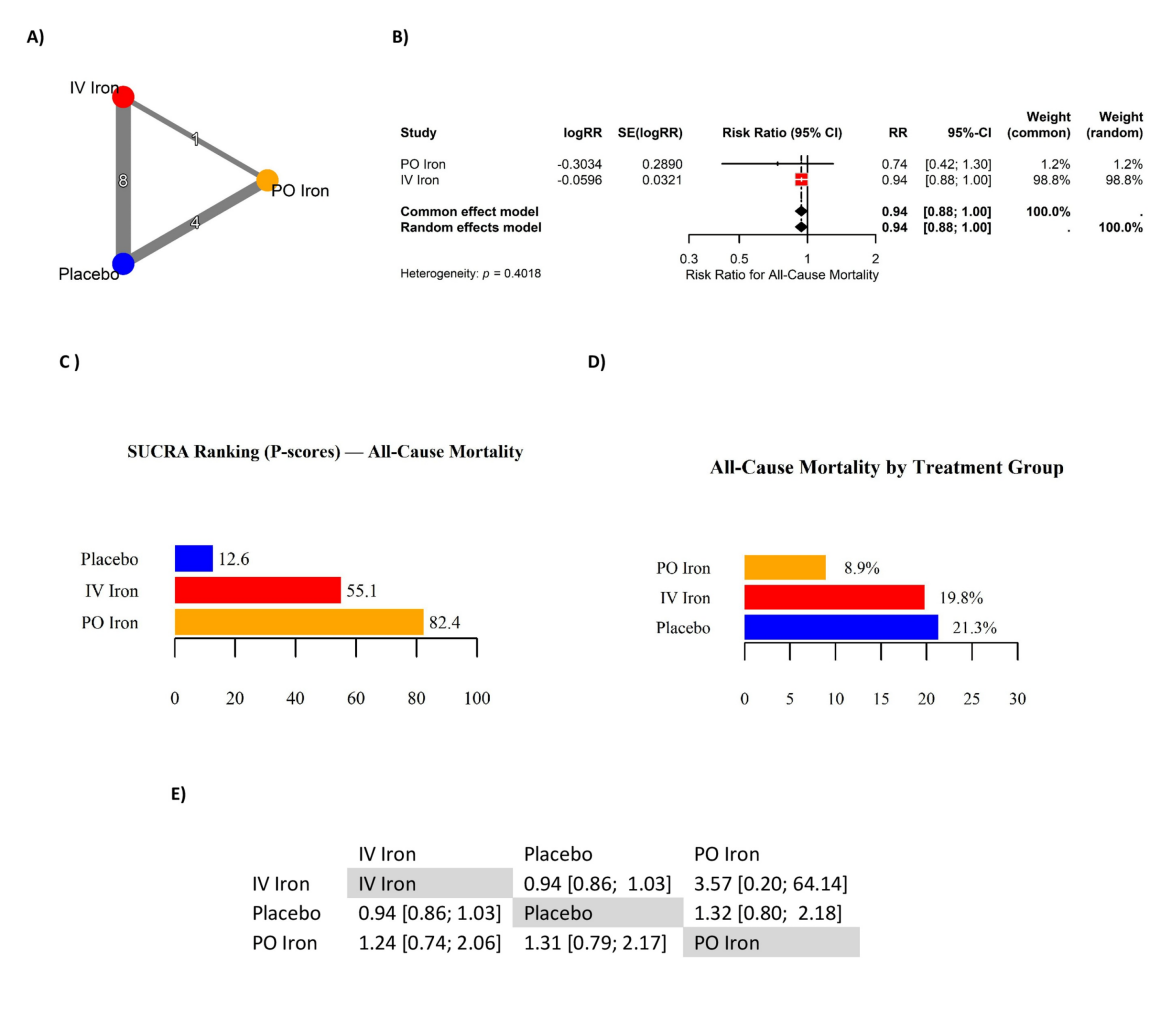
Network meta-analysis of all-cause mortality in iron-deficient heart failure. (A) Network geometry; (B) forest plot of risk ratios (RRs); (C) SUCRA rankings (P-scores) for all-cause mortality; (D) absolute event rates by treatment group; (E) league table of pairwise RRs. References [10,2,4,11,5,6,12,13,15,7,16,17] CI: confidence interval; IV: intravenous; PO: per os (oral); SUCRA: surface under the cumulative ranking

Twelve RCTs contributed to the NMA evaluating the effect of IV and PO iron on all-cause mortality in patients with HF and ID (Table 6). The network geometry (Figure 5A) included eight trials comparing IV iron with placebo, four comparing PO iron with placebo, and one trial directly comparing IV and PO iron. Pooled RRs showed that IV iron was associated with a non-significant reduction in all-cause mortality compared to placebo (RR: 0.94, 95% CI: 0.88 to 1.00) (Figure 5B). PO iron also yielded a non-significant effect (RR: 0.74, 95% CI: 0.42 to 1.30), based on limited and imprecise evidence. The overall pooled estimate (RR: 0.94, 95% CI: 0.88 to 1.00) trended toward benefit, though not statistically significant. SUCRA-based treatment rankings (Figure 5C) suggested PO iron had the highest probability of being the most effective intervention in reducing mortality (82.4), followed by IV iron (55.1) and placebo (12.6). However, this ranking reflects point estimates from sparse and highly imprecise trials, and should be interpreted cautiously. The direct IV vs. PO comparison was extremely imprecise (RR: 3.57, 95% CI: 0.20 to 64.14) (Figure 5E), reflecting limited sample size rather than a true treatment effect. Follow-up duration ranged from 12 weeks to 2.7 years. Collectively, neither IV nor PO iron demonstrated a statistically significant survival benefit. While SUCRA values numerically favored PO iron, the wide CIs and sparse event data preclude firm conclusions. These findings highlight the need for adequately powered trials to determine whether iron therapy influences mortality outcomes in HF.

**Table 6:** All-cause and cardiovascular mortality by treatment arm in included trials. IV: intravenous; CV: cardiovascular; PO: per os (oral)

Study	Arm	N	All-Cause Deaths (n)	Cardiovascular Deaths (n)	Follow-Up
FERRIC-HF (2008) [10]	IV Iron	24	1	0	18 weeks
	Placebo	11	0	0	18 weeks
FAIR-HF (2009) [2]	IV Iron	304	5	4	24 weeks
	Placebo	155	4	4	24 weeks
CONFIRM-HF (2015) [4]	IV Iron	152	12	11	52 weeks
	Placebo	152	14	12	52 weeks
EFFECT-HF (2017) [11]	IV Iron	88	0	0	24 weeks
	Placebo	86	4	1	24 weeks
AFFIRM-AHF (2020) [5]	IV Iron	567	77	61	52 weeks
	Placebo	565	87	70	52 weeks
IRONMAN (2022) [6]	IV Iron	569	184	119	Median 2.7 years
	Placebo	568	193	138	Median 2.7 years
HEART-FID (2023) [12]	IV Iron	1532	361	251	52 weeks
	Placebo	1533	376	275	52 weeks
IRON-HF (2013) [13]	IV Iron	10	2	2	12 weeks
	PO Iron	7	0	0	12 weeks
	Placebo	6	2	1	12 weeks
Zdravkovic et al. (2019) [15]	PO Iron	100	9	NR	24 weeks
	Placebo	101	17	NR	24 weeks
IRONOUT-HF (2017) [7]	PO Iron	111	14	14	16 weeks
	Placebo	114	12	12	16 weeks
Suryani et al. 2016 [16]	PO Iron	27	1	NR	12 weeks
	Placebo	27	2	NR	12 weeks
SI-HF (2021) [17]	PO Iron	25	0	0	24 weeks
	Placebo	25	0	0	24 weeks

CV Mortality

**Figure 6:**
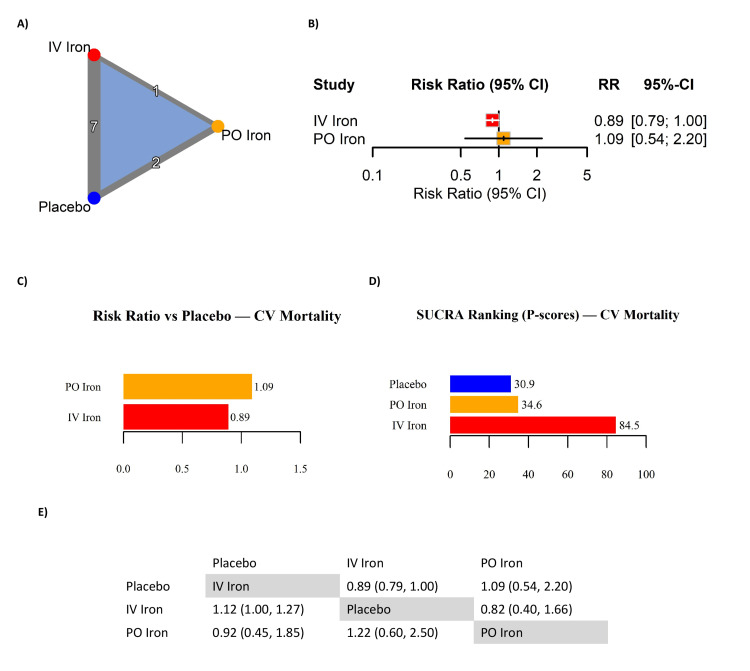
Network meta-analysis of cardiovascular (CV) mortality in iron-deficient heart failure. (A) Network geometry; (B) forest plot of risk ratios (RRs); (C) RRs versus placebo for CV mortality; (D) SUCRA rankings (P-scores) for CV mortality; (E) league table of pairwise RRs. References [10,2,4,11,5,6,12,13,7,16,17] CI: confidence interval; IV: intravenous; PO: per os (oral); SUCRA: surface under the cumulative ranking

Ten RCTs were included in the NMA evaluating the effect of IV and PO iron on CV mortality in patients with HF and ID (Table 6). The network geometry (Figure 6A) included seven IV iron vs. placebo comparisons, two PO iron vs. placebo comparisons, and one direct IV vs. PO trial, forming a fully connected triangular network. Pooled estimates (Figure 6B) indicated a non-significant reduction in CV mortality with IV iron vs. placebo (RR: 0.89, 95% CI: 0.79 to 1.00), narrowly missing statistical significance. In contrast, PO iron showed no evidence of benefit (RR: 1.09, 95% CI: 0.54 to 2.20) with wide CIs reflecting sparse event data and imprecision. Visualized estimates (Figure 6C) were consistent with this trend, favoring IV iron over both placebo and PO iron. Treatment rankings based on SUCRA scores (Figure 6D) suggested IV iron had the highest probability of being most effective in reducing CV mortality (SUCRA = 84.5), followed by PO iron (34.6) and placebo (30.9). However, these rankings should be interpreted with caution, as they are based on limited PO iron data with wide CIs. The indirect IV vs. PO comparison (RR: 0.82, 95% CI: 0.40 to 1.66) (Figure 6E) was consistent with a trend favoring IV iron but did not achieve statistical significance. CV mortality analyses were based on binary events at trial end. Follow-up duration varied from 12 weeks to 2.7 years. In summary, IV iron demonstrated a consistent but non-significant trend toward reducing CV mortality, while PO iron did not show a clear benefit and remained highly imprecise. The evidence suggests potential for IV iron in reducing CV deaths, but adequately powered trials are needed to confirm this effect.

Serum Iron Indices: Ferritin and TSAT

**Figure 7:**
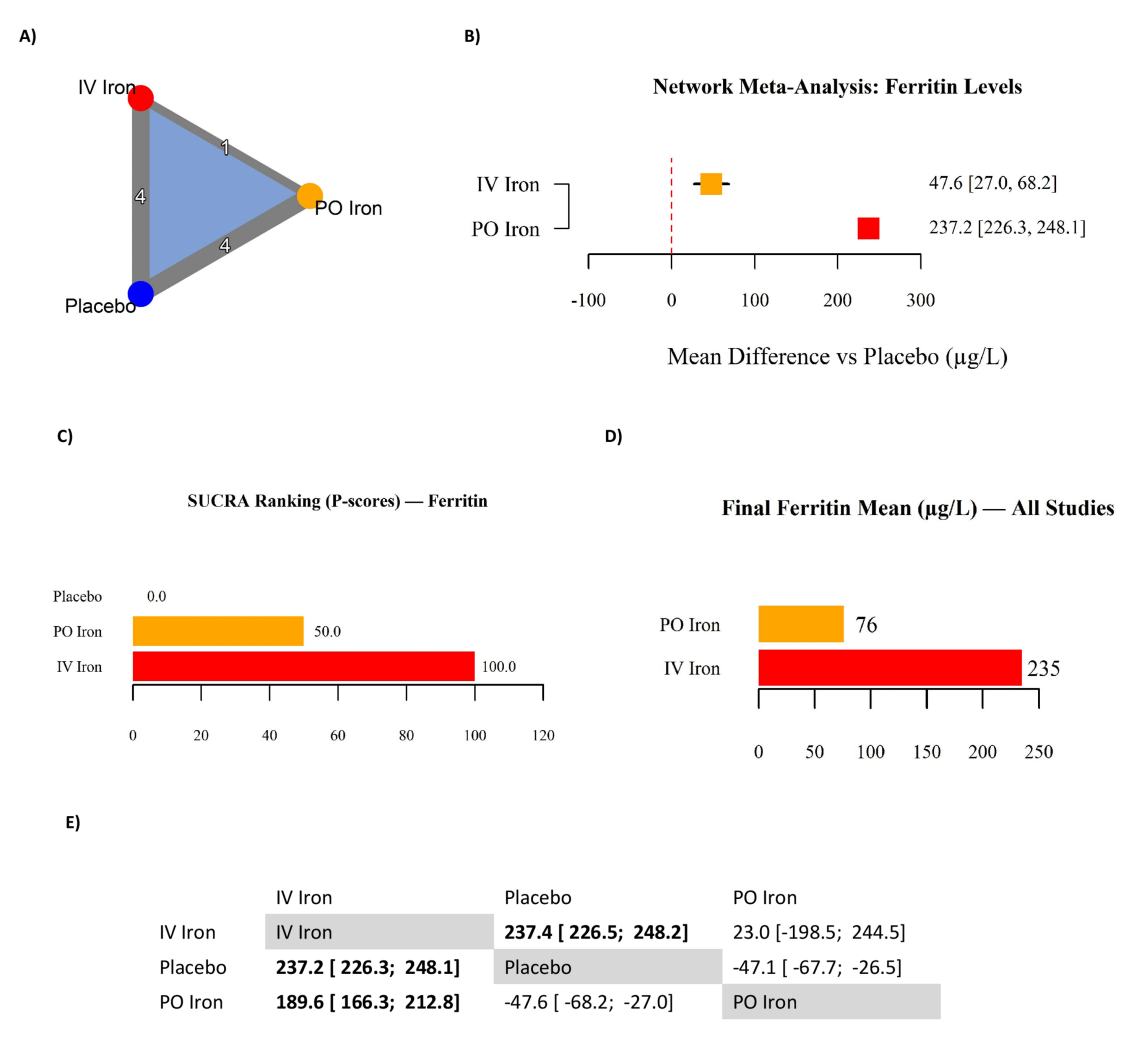
Network meta-analysis of serum ferritin levels in iron-deficient heart failure. (A) Network geometry; (B) forest plot of mean differences (MDs) versus placebo (µg/L); (C) SUCRA rankings (P-scores) for ferritin; (D) final mean ferritin levels (µg/L) across all studies; (E) league table of pairwise MDs. References [10,2,4,5,12,13,15-17] IV: intravenous; PO: per os (oral); TSAT: transferrin saturation; SUCRA: surface under the cumulative ranking; CI: confidence interval

Ferritin: Nine RCTs reporting post-treatment ferritin levels were included in the NMA (Table 7). The network geometry (Figure 7A) demonstrated strong connectivity across IV iron, PO iron, and placebo. Both formulations significantly increased ferritin compared to placebo. IV iron produced the largest effect (MD: 237.2 µg/L; 95% CI: 226.3 to 248.1), while PO iron achieved a smaller but significant effect (MD: 47.6 µg/L; 95% CI: 27.0 to 68.2) (Figure 7B). Absolute post-treatment means (Figure 7D) corroborated these findings, with IV iron achieving markedly higher ferritin levels (mean: 235 µg/L) compared to PO iron (76 µg/L). SUCRA-based rankings (Figure 7C) confirmed IV iron as most effective (100.0), followed by PO iron (50.0) and placebo (0.0). Indirect comparisons (Figure 7E) reinforced these results, showing IV iron significantly superior to PO iron (MD: 189.6 µg/L; 95% CI: 166.3 to 212.8). Trials reporting medians and IQRs could not be converted and were excluded from pooling, though their findings were directionally consistent. These results demonstrate that IV iron provides a substantially greater and more consistent increase in ferritin, reflecting its superior ability to restore storage iron.

**Table 7:** Summary of ferritin and transferrin saturation (TSAT) outcomes by treatment arm across included trials. IV: intravenous; PO: per os (oral); SD: standard deviation; IQR: interquartile range; SUCRA: surface under the cumulative ranking

Study	Arm	N	ΔFerritin (ng/mL), Mean (SD)	ΔTSAT (%), Mean (SD)	Follow-Up
FERRIC-HF (2008) [10]	IV Iron	24	324 ± 189	13 ± 9	18 weeks
	Placebo	11	51 ± 85	2 ± 7	18 weeks
FAIR-HF (2009) [2]	IV Iron	304	246 ± 20	11.3 ± 12.3	24 weeks
	Placebo	155	13.9 ± 66.9	2.3 ± 8.2	24 weeks
CONFIRM-HF (2015) [4]	IV Iron	152	200 ± 19	5.7 ± 1.2	52 weeks
	Placebo	152	Negligible	Negligible	52 weeks
EFFECT-HF (2017) [11]	IV Iron	88	NR	9.7 ± 9.5	24 weeks
	Placebo	86	NR	2.1 ± 9.5	24 weeks
AFFIRM-AHF (2020) [5]	IV Iron	569	444 (median)	10 (median)	Median 2.7 years
	Placebo	568	NR	NR	Median 2.7 years
HEART-FID (2023) [12]	IV Iron	1532	244 ± 190	7.7 ± 9	52 weeks
	Placebo	1533	2.5 ± 6	2.5 ± 12	52 weeks
IRON-HF (2013) [13]	IV Iron	10	126	10 ± 11.5	12 weeks
	PO Iron	7	103	5 ± 10.2	12 weeks
	Placebo	6	-42	2 ± 6.9	12 weeks
Zdravkovic et al. (2019) [15]	PO Iron	100	28.11 ± 24.02	21	24 Weeks
	Placebo	101	29.32 ± 28.08	23.4	24 weeks
IRONOUT-HF (2017) [7]	PO Iron	111	18 (-8 to 38) (median)	2 (-3 to 7) (median)	16 weeks
	Placebo	114	1 (-15 to 17) (median)	NR	16 weeks
Suryani et al. 2016 [16]	PO Iron	27	86 ± 126	13.9 ± 10.1	12 weeks
	Placebo	27	2 ± 92	3 ± 11	12 weeks
SI-HF (2021) [17]	PO Iron	25	39.89 ± 46.67	NR	24 weeks
	Placebo	25	-0.91 ± 9.92	NR	24 weeks

**Figure 8:**
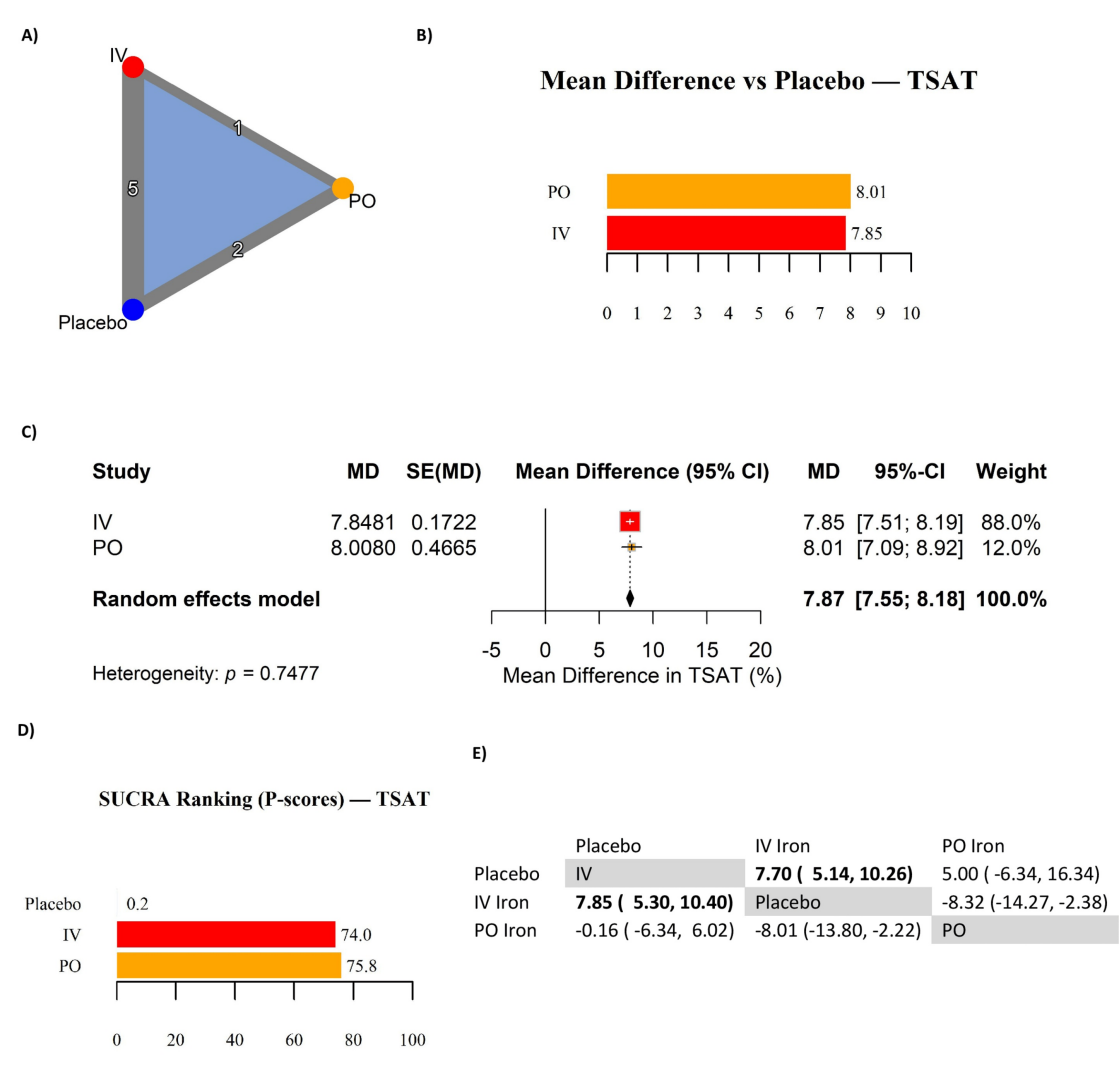
Network meta-analysis of transferrin saturation (TSAT) in iron-deficient heart failure. (A) Network geometry; (B) mean differences (MDs) versus placebo in TSAT (%); (C) forest plot of MDs with 95% confidence intervals (CIs); (D) SUCRA rankings (P-scores) for TSAT; (E) league table of pairwise MDs. References [10,2,4,11-17] IV: intravenous; PO: per os (oral); SUCRA: surface under the cumulative ranking

TSAT: Eight RCTs contributed to the analysis of TSAT outcomes. Both IV and PO iron significantly improved TSAT compared to placebo (Table 7). The pooled effect was 7.85% (95% CI: 7.51 to 8.19) for IV iron and 8.01% (95% CI: 7.09 to 8.92) for PO iron (Figures 8B-8C), with no evidence of heterogeneity (p = 0.75). SUCRA rankings (Figure 8D) showed similar probabilities for IV iron (74.0) and PO iron (75.8), both far exceeding placebo (0.2). The indirect comparison (Figure 8E) confirmed no significant difference between IV and PO iron (MD: -0.16%; 95% CI: -6.34 to 6.02). Trials that did not report TSAT values (NR) were excluded from this analysis to avoid bias.

Taken together, the findings indicate that while both IV and PO iron significantly improve iron indices compared to placebo, IV iron provides a markedly larger increase in ferritin and thus more robust correction of storage ID, whereas both formulations achieve similar gains in TSAT, reflecting comparable improvements in functional iron availability.

Summary of Treatment Rankings and Comparative Efficacy

IV iron therapy emerges as the top intervention for most endpoints: Best at replenishing iron stores (ferritin, TSAT), reducing HF hospitalization risk (high-quality evidence), provides clear improvement in exercise capacity (moderate evidence), and may reduce CV mortality slightly (moderate evidence, borderline significance).

PO iron therapy: Improves exercise capacity in some studies (especially newer formulations), but overall evidence is less consistent (moderate certainty, benefit not guaranteed); only modestly raises iron markers, often insufficient to fully correct the deficiency; and has not shown a significant effect on clinical outcomes (no clear reduction in hospitalizations or mortality).

Placebo/standard care obviously ranks lowest, as expected, with continued ID linked to ongoing functional impairment (Table 8).

**Table 8:** GRADE summary of findings for key outcomes assessed in the network meta-analysis. Effect sizes >0 favor iron therapy for six-minute walk distance (6MWD), ferritin, and transferrin saturation (TSAT); risk ratios (RRs) <1 favor iron therapy for clinical events. Certainty of evidence rated using the GRADE framework, adapted for network meta-analysis [19]. References [2,4-7,10-17] CI: confidence interval; GRADE: Grading of Recommendations Assessment, Development and Evaluation

Outcome	Comparison	Effect Estimate (95% CI)	Interpretation	Evidence Certainty
6MWD (m)	IV iron vs. Placebo	26.02 m (17.85 to 34.90)	IV iron improves 6MWD (statistically significant)	Moderate
	PO iron vs. Placebo	35.1 m (-11 to +81)	PO Iron ranked best, but is not statistically significant	Moderate
	IV iron vs. PO iron	-9.1 m (-60 to +42)	No clear difference	Moderate
HF Hospitalization	IV iron vs. Placebo	RR 0.79 (0.66 to 0.93)	21% relative risk reduction (statistically significant)	High
	PO iron vs. Placebo	RR 0.83 (0.67 to 1.03)	Trend to fewer events, not significant	Low
	IV iron vs. PO iron	RR 0.80 (0.48 to 1.34)	No clear difference (favours IV, not significant)	Low
All-cause Mortality	IV iron vs. Placebo	RR 0.94 (0.86-1.00)	No significant difference (trend to benefit)	Low
	PO iron vs. Placebo	RR 0.74 (0.42-1.30)	Uncertain (few events)	Low
	IV iron vs. PO iron	RR 1.27 (0.60-2.68)	PO better but not statistically significant	Low
CV Mortality	IV iron vs. Placebo	RR 0.89 (0.79-1.00)	Borderline reduction (11%) (not statistically significant)	Moderate
	PO iron vs. Placebo	RR ~0.8 (wide CI)	Uncertain, wide CI (not statistically significant)	Low
	IV iron vs. PO iron	RR 0.82 (0.40 to 1.66)	IV iron may reduce but not statistically significant	Low
Ferritin (μg/L)	IV iron vs. Placebo	237.2 µg/L (226.3 to 248.1)	Greatly increases ferritin (statistically significant)	High
	PO iron vs. Placebo	47.6 µg/L (27.0 to 68.2)	moderately increases (statistically significant)	Moderate
	IV iron vs. PO iron	189.6 µg/L (166.3 to 212.8)	IV iron is significantly superior to PO iron	Moderate
TSAT (%)	IV iron vs. Placebo	7.85% (95% CI: 7.51 to 8.19)	Corrects iron deficiency (statistically significant)	High
	PO iron vs. Placebo	8.01% (95% CI: 7.09 to 8.92)	Comparable correction (statistically significant)	Moderate
	IV iron vs. PO iron	-0.16% (95% CI: -6.34 to 6.02)	PO better but not statistically significant	Low

Discussion

This systematic review and NMA provide the first comprehensive, head-to-head comparison of IV vs. PO iron supplementation in iron-deficient HF, integrating data from 13 trials. The findings offer several important insights.

Improvement in Functional Capacity

Both IV and PO iron can improve exercise tolerance in HF, as reflected by increases in 6MWD and self-reported symptoms. IV iron’s benefits on 6MWD are well established (roughly +30 m on average) [4], and we confirmed that benefit with moderate certainty. PO iron, in aggregate, showed a similar point estimate, suggesting that if adequately absorbed and over sufficient time, PO iron might also improve functional status. In fact, the two small trials that showed large 6MWD gains with PO iron used high doses or novel formulations (ferrous sulfate 600 mg daily and sucrosomial iron) in patients who likely had severe deficiency and/or anemia at baseline [16]. This indicates that the principle of iron repletion improving exercise capacity holds true regardless of route, but the challenge is that PO iron often fails to replete iron in HF (due to hepcidin-driven malabsorption and intolerance). The IRONOUT-HF investigators noted that despite high-dose PO iron for four months, iron stores barely budged, and no functional improvement occurred [7]. Thus, in practice, IV iron is far more reliable for achieving the iron repletion necessary to improve exercise capacity. Our results do not support dismissing PO iron entirely, especially newer forms like sucrosomial iron, which might have better uptake; however, the consistency and magnitude of benefit clearly favor IV iron.

HF Hospitalization and Clinical Status

One of the most striking results is the robust reduction in HF hospitalizations with IV iron (RR 0.79, 95% CI: 0.66-0.93, representing a ~21% relative reduction). This corresponds to an absolute risk reduction of ~5-6% at 12 months, with an estimated number needed to treat (NNT) of ~18-20 patients to prevent one HF hospitalization. This aligns with prior meta-analyses and the conclusions of trials like CONFIRM-HF and AFFIRM-AHF that treating ID leads to fewer HF decompensations [4,5]. Mechanistically, correcting ID may improve skeletal muscle function and efficiency, thereby reducing symptomatic deterioration and breaking the cycle of frailty and HF exacerbation. The fact that AFFIRM-AHF (in acute HF) and IRONMAN (chronic HF) both pointed in this direction, and our NMA solidifies it, is important for clinicians [5,6]. It means IV iron is not just a symptomatic therapy but a disease-modifying therapy in HFrEF in terms of reducing rehospitalizations. This outcome is of high relevance to patients and health systems. Although our NMA could not stratify anemia vs. isolated ID, subgroup analyses from FAIR-HF and IRONMAN suggest that the benefit of IV iron extends to both groups, supporting ID itself as the key therapeutic target. On the other hand, PO iron did not show such an effect - again likely because of insufficient iron repletion. It’s worth noting that in chronic HF management, timing and patient selection for iron therapy matter: patients with very low ferritin/TSAT and worse functional class appear to benefit most, as seen in trial subgroup analyses [4]. Nonetheless, given the high safety profile of IV iron, treating ID in HF appears to yield substantial reductions in morbidity. Our findings support current guideline recommendations (class IIa) to use IV iron in HFrEF with ID to improve outcomes.

Mortality and Long-Term Outcomes

We did not find a clear mortality reduction with IV iron in the overall analysis, consistent with recent trial evidence [12]. There are a few considerations here. First, most trials were not powered for mortality; of the two that were (HEART-FID and IRONMAN), both were impacted by the COVID-19 pandemic and did not meet their primary endpoints for mortality/HF hospitalization composites [6,12]. Interestingly, IRONMAN’s post-hoc analysis excluding COVID-19-related disruptions did show a significant benefit in the primary composite (HF hospitalization or CV death) [6]. This hints that with more follow-up or under less confounded conditions, a mortality benefit might emerge. Second, iron therapy may need more time to translate into survival gains, beyond the follow-up of current trials. Third, it’s possible that iron repletion mainly affects QOL and hospitalization risk, and not the underlying disease progression enough to alter mortality (especially as modern HFrEF patients are on multiple other therapies that drive survival, potentially overshadowing a smaller incremental effect of iron). This also helps reconcile why large gains in ferritin and TSAT did not translate directly into mortality reductions: these biomarkers reflect correction of ID and improvements in functional status, but mortality is a harder endpoint influenced by multiple competing risks. In any case, our analysis suggests caution in claiming mortality benefit at this point. The ongoing FAIR-HF2 trial will shed light on this, as it focuses on hard outcomes over longer follow-up. Until then, clinicians should discuss with patients that IV iron is primarily to help them feel better and stay out of the hospital, and any survival advantage remains unproven.

PO Iron - Is There a Role?

Based on our findings, PO iron cannot be considered a substitute for IV iron in symptomatic HFrEF with true ID. However, PO iron might be better than nothing in cases where IV iron is not accessible. For instance, in low-resource settings or patient refusal of infusions, a trial of PO iron (preferably a well-tolerated form like sucrosomial or a low-dose regimen to reduce side effects) could be attempted, and if iron indices improve and the patient feels better, that’s a win. But if there’s no response in iron labs or symptoms, one should promptly escalate to IV iron. Another potential niche for PO iron is long-term maintenance after initial IV repletion. Future research should explicitly test sequential therapy strategies (e.g., initial IV repletion followed by PO maintenance), as current evidence is insufficient. A hypothesis is that after ferritin is raised with IV iron, PO supplements might maintain iron stores, though HF patients’ ongoing inflammation may still impede PO absorption. Our analysis did not directly address this strategy, as trials were not designed that way. It’s an area for future research (some studies are exploring combination or sequential approaches).

Safety and Tolerability

Although not the primary focus of our analysis, safety outcomes reported in trials were generally favorable. IV iron did not increase the risk of adverse events significantly - hypersensitivity reactions were rare (<1 in 200 patients) and no iron overload issues were noted (ferritin levels peaked within a safe range). PO iron’s main drawback was GI side effects (constipation, nausea), leading to discontinuation in a minority (e.g., ~5%-10%). In one trial of PO iron, ~17% of patients had GI side effects, though serious adverse events were similar to placebo. Known concerns, such as infection risk or hypophosphatemia, were not systematically reported across trials; however, large RCTs did not demonstrate excess infections, and ferric derisomaltose may have a lower hypophosphatemia risk compared to ferric carboxymaltose. Taken together, IV iron is a relatively safe therapy in HF when guideline criteria are respected (avoiding use in active infection, very high ferritin, etc.), and PO iron is also safe but less well tolerated.

Quality of Evidence

We found high-quality evidence for the IV iron benefits on functional and hospital outcomes, but lower quality for PO iron comparisons. Some risk of bias in smaller trials and imprecision lowered confidence in the PO iron findings. The NMA approach allowed us to use all available information, but reliance on indirect comparisons (e.g., IV vs. PO) also introduces some uncertainty. Still, the overall consistency of results across trials lends credibility. SUCRA rankings were incorporated into interpretation cautiously: IV iron consistently ranked highest for hospitalization and ferritin with high or moderate certainty, whereas PO iron often ranked second but with low certainty due to sparse and imprecise data. Our review underscores the need for continued high-quality research: for example, more direct comparisons of modern PO iron formulations vs. IV iron would be informative (to see if any PO strategy can approach the efficacy of IV). Iron repletion offers an additive benefit on top of background therapies - current data suggests yes, since trials included patients on good background therapy [20].

Limitations of This Review

By design, our NMA inherits the limitations of the included trials. There was variability in patient populations. We combined them assuming a class effect of iron repletion in HFrEF regardless of timing, but there could be scenario-specific differences (e.g., acute HF patients may derive more hospitalization benefit acutely, as seen in AFFIRM-AHF [5]). We attempted to check for inconsistency; none was significant, supporting our approach. Another limitation is the lack of data in HF with preserved EF (HFpEF) - all trials were in HFrEF except a small subset in FAIR-HF with mid-range EF [2]. So our conclusions apply to HFrEF; it’s unknown if HFpEF patients respond similarly (studies like FAIR-HFpEF are underway). We also did not explicitly analyze quality-of-life scores due to heterogeneity, but narrative data consistently show IV iron improves QOL (KCCQ scores +5 to +10 points) [4]. Finally, while we harmonized data across different follow-up durations, formulations, and doses, this heterogeneity may still affect generalizability.

Clinical Implications

For clinicians, this analysis reinforces that IV iron should be strongly considered in symptomatic HFrEF patients with ID (ferritin <100 or <300 with TSAT <20%). It can lead to moderate improvements in exercise capacity (patients often report feeling less fatigued, able to walk farther) and, importantly, fewer HF hospitalizations - a benefit that can manifest within three to six months of therapy. The typical regimen is ferric carboxymaltose 500 mg IV given two to three times over a few weeks, or a single 1000 mg ferric derisomaltose infusion, with re-dosing every four to six months as needed based on labs. This approach is now part of routine HF management in many centers.

PO iron cannot be recommended as an equivalent alternative when these outcome benefits are desired. It may serve as a stopgap or adjunct. Our findings might also guide patient discussions - for instance, if a patient is needle-averse and insists on trying PO supplements first, the clinician can explain that while PO iron might help incrementally, the evidence suggests IV iron provides a more reliable and larger benefit in HF. At a system level, while IV iron is more resource-intensive than PO therapy, reductions in rehospitalizations may make it cost-effective in many settings, though accessibility remains a challenge in low-resource environments.

## Conclusions

Our systematic review and NMA demonstrate that in patients with HF and ID, IV iron therapy (particularly ferric carboxymaltose or ferric derisomaltose) provides clinically meaningful benefits. These include a ~26 m improvement in 6MWD and a ~21% relative reduction in HF hospitalizations (RR 0.79, 95% CI: 0.66-0.93), supported by moderate to high certainty of evidence. IV iron is also superior to PO iron in restoring ferritin and iron stores. PO iron showed only modest improvements in iron parameters and inconsistent gains in functional capacity, with no clear effect on hospitalizations or mortality. Its role should be considered limited to situations where IV iron is not feasible (e.g., resource-constrained settings or patient refusal). From a practical standpoint, clinicians should actively screen HF patients for ID by checking ferritin and TSAT and promptly correct it, primarily with IV iron, as part of guideline-directed HF management. Follow-up monitoring of ferritin and TSAT is essential to guide re-dosing intervals (typically every four to six months). This therapy is generally safe and well-tolerated, with low rates of hypersensitivity and no excess in serious adverse events reported in large RCTs.

Importantly, these conclusions apply primarily to patients with HFrEF, as nearly all included trials enrolled reduced-EF populations; the role of iron therapy in HFpEF remains uncertain. While there is no definitive evidence yet that IV iron extends survival, its demonstrated benefits on morbidity, functional status, and QOL make it a valuable intervention in HF care. Future research should clarify long-term mortality outcomes, examine potential benefits in HFpEF, and explore whether newer PO formulations or sequential strategies (IV repletion followed by PO maintenance) can broaden therapeutic options. For now, however, IV iron remains the evidence-based standard of care for correcting ID in HFrEF.

## Appendices

Appendix A

The full search strategy is outlined as follows:

Databases searched: PubMed/MEDLINE, Embase, and Cochrane Central Register of Controlled Trials (CENTRAL).

Date of last search: March 15, 2025.

Filters applied: None (no language or date restrictions).

Grey literature: Not included.

PubMed (MEDLINE)

("heart failure"[MeSH Terms] OR "cardiac failure" OR "congestive heart failure" OR "HFrEF" OR "HFpEF") AND ("iron"[MeSH Terms] OR "iron supplementation" OR "iron therapy" OR "ferric carboxymaltose" OR "ferric derisomaltose" OR "iron sucrose" OR "ferrous sulfate" OR "ferrous fumarate" OR "sucrosomial iron" OR "ferric polymaltose" OR "oral iron" OR "intravenous iron") AND ("randomized controlled trial"[Publication Type] OR "randomized"[Title/Abstract] OR "RCT"[Title/Abstract])

Embase

('heart failure'/exp OR 'cardiac failure' OR 'congestive heart failure' OR HFrEF OR HFpEF) 

AND ('iron supplementation'/exp OR 'iron therapy' OR 'ferric carboxymaltose' OR 'ferric derisomaltose' OR 'iron sucrose' OR 'ferrous sulfate' OR 'ferrous fumarate' OR 'sucrosomial iron' OR 'ferric polymaltose' OR 'oral iron' OR 'intravenous iron') 

AND ('randomized controlled trial'/exp OR randomized:ab,ti OR RCT:ab,ti)

Cochrane Central (CENTRAL)

("heart failure" OR "cardiac failure" OR "congestive heart failure" OR HFrEF OR HFpEF) AND ("iron" OR "iron supplementation" OR "iron therapy" OR "ferric carboxymaltose" OR "ferric derisomaltose" OR "iron sucrose" OR "ferrous sulfate" OR "ferrous fumarate" OR "sucrosomial iron" OR "ferric polymaltose" OR "oral iron" OR "intravenous iron") IN Trials

Additional Measures

ClinicalTrials.gov and WHO ICTRP were searched manually to confirm trial eligibility and identify unpublished or ongoing studies. Duplicate publications were cross-checked by trial registry identifiers.

Appendix B

**Table 9:** PRISMA 2020 checklist PRISMA: Preferred Reporting Items for Systematic Reviews and Meta-Analyses

Section/Topic	PRISMA Item	Location in Manuscript
Title	Identify report as systematic review/meta-analysis	Title page
Abstract	Structured summary	Abstract
Rationale	Rationale for review	Introduction, para 1-2
Objectives	Explicit statement of objectives	Introduction, last para
Eligibility	Criteria for including/excluding studies	Methods, Eligibility Criteria
Information sources	Databases and dates searched	Methods, Search Strategy; Appendix 1
Search strategy	Full search strings	Appendix 1
Selection process	Screening and selection methods	Methods, Study Selection
Data collection	Extraction process	Methods, Data Extraction and Handling
Risk of bias	Method of RoB assessment	Methods, Risk of Bias Assessment
Effect measures	Effect size metrics	Methods, Statistical Analysis
Synthesis methods	Statistical approach and models	Methods, Statistical Analysis
Heterogeneity	How heterogeneity was assessed	Methods, Statistical Analysis
Reporting bias	How publication bias was addressed	Methods, Statistical Analysis
Certainty assessment	GRADE approach	Methods, Certainty of Evidence
Study characteristics	Summary of included trials	Results, Table 1
Results of individual studies	Outcomes and estimates	Results, Figures/Tables
Synthesis of results	Pooled effect estimates	Results, main text
Risk of bias across studies	Summary of study-level RoB	Results, Risk of Bias subsection
Discussion	Summary of findings, limitations	Discussion
Limitations	Study and review-level limitations	Discussion
Conclusions	Interpretation and implications	Discussion, Conclusions
Registration	Protocol registration (if applicable)	Methods, first paragraph
Support	Funding and support	Funding statement
Competing interests	Disclosures	Conflict of interest section
Availability of data	Data and code availability	Data availability statement

Appendix C

Risk of Bias: Expanded Methods

Tool and domains: We used the Cochrane RoB 2.0 tool, covering randomization process, deviations from intended interventions, missing outcome data, outcome measurement, and reporting bias.

Reviewer process: Two reviewers independently rated each trial. Disagreements were resolved by discussion and, if unresolved, by a third senior reviewer.

Criteria

Low risk: All domains clearly reported and appropriate.

Some concerns: Incomplete or unclear reporting in ≥1 domain.

High risk: Evidence of methodological limitations likely to bias results.

Reliability: Inter-rater reliability (κ) was not formally calculated, but initial agreement exceeded 90%.

Sensitivity analysis: Down-weighting or excluding high-risk trials did not materially change the direction or statistical significance of the results.

Influence of high-risk trials: No single high-risk trial disproportionately influenced outcomes; effects remained consistent across key endpoints.

Appendix D​​

The following are the expanded statistical methods:

Assumptions and Model Structure

Transitivity: Treatment effects were assumed to be transitive across comparisons because included RCTs recruited broadly similar populations (predominantly HFrEF with iron deficiency defined by ferritin <100 μg/L or 100-300 μg/L with TSAT <20%) and applied comparable interventions in similar clinical settings. Indirectness from transitivity violations was considered explicitly in GRADE.

Heterogeneity: A common heterogeneity parameter (τ^2^) was used across all treatment contrasts, in line with standard NMA methodology, to allow borrowing of strength across comparisons.

Multi-arm Trials

Multi-arm trials (e.g., IV vs. PO vs. placebo) were handled by combining arms at the node level (e.g., pooling PO formulations), which preserves randomization and avoids unit-of-analysis errors or double-counting of controls.

Small-Study Effects and Publication Bias

Because most contrasts included <10 RCTs, Egger’s regression and Peters’ test were not feasible. We inspected comparison-adjusted funnel plots for the network and contour-enhanced funnel plots for the largest contrast (IV vs. placebo). Potential small-study effects were considered qualitatively when grading the certainty of evidence.

Sensitivity Analyses

We repeated analyses using fixed-effect versus random-effects models. We also performed sensitivity analyses excluding high-risk-of-bias trials. Neither materially altered the direction or significance of the results.

Normality and Continuous Outcomes

Continuous outcomes (six-minute walk distance (6MWD), ferritin, transferrin saturation (TSAT)) were reported as approximately normally distributed in included trials. No data transformations were required.

Missing Data Handling

Trials reporting only medians and interquartile ranges were excluded from quantitative synthesis and described narratively. Missing SDs were imputed from reported baseline or arm-level SDs when feasible; if not possible, the trial was excluded from that analysis.

Follow-Up Harmonization

Outcomes were harmonized by extracting the longest available follow-up between 3 and 12 months for the main analysis. Trials with longer follow-up (e.g., IRONMAN, up to 2.7 years) were included in sensitivity analyses to test robustness.

SUCRA Rankings

SUCRA values were calculated for all outcomes. To avoid misinterpretation, SUCRA rankings were presented alongside effect estimates and certainty ratings, rather than as evidence of clinical superiority.

Prediction Intervals

For pairwise meta-analyses, 95% prediction intervals were calculated to provide an estimate of the range of effects expected in future studies.
